# Unusual presentation of orbital cysticercosis-ptosis, diminution of vision and medial rectus weakness: a case report

**DOI:** 10.4076/1757-1626-2-7025

**Published:** 2009-08-12

**Authors:** Bharati Taksande, Ulhas Jajoo, Samir Yelwatkar, Jaikishan Ashish

**Affiliations:** Department of Medicine, Mahatma Gandhi Institute of Medical SciencesSevagram, District, Wardha - 442102, MaharashtraIndia

## Abstract

Cysticercosis is the most common parasitic disease of the nervous system. The disease occurs when humans become the intermediate host in the life cycle of *Taenia solium* by ingesting its eggs from contaminated food. The most common sites of involvement of cysticerci are soft tissue, eye and central nervous system. Unusual location of the cysts may result in uncommon manifestations. Ocular cysticercosis can involve both the intraocular and extra ocular muscle. Extra ocular muscle cysticercosis is rare. We are reporting the unusual manifestation of ptosis, proptosis, diminution of vision and medial rectus palsy due to cysticercosis. The patient was successfully treated with systemic steroids and albendazole.

## Introduction

Cysticercosis is the most common parasitic disease of the nervous system. The disease occurs when humans become the intermediate host in the life cycle of Taenia solium by ingesting its eggs from contaminated food. The cysts of cysticercus cellulosae in man lodge frequently in muscles, central nervous system and in the eye [1]. Unusual location of the cysts may result in uncommon manifestations mimicking a host of neurological disorders [2].

Ocular cysticercosis involve both the intraocular and extra ocular muscle. Extra ocular muscle cysticercosis is rare. Unusual presentations of extra ocular muscle are mentioned in the literature. We here are reporting a unusual manifestation of ptosis, proptosis, diminution of vision and medial rectus palsy due to cysticercosis.

## Case presentation

A 40-year-old man, origin of south-east Asian country from India presented to the medicine ward with complaints of drooping of left eyelid and diminution of vision in the same eye from 7 days. The drooping of eyelid was associated with pain. The pain was aggravated by movement of the eyeball. He had a history of intermittent protrusion of left eyeball since one year. There was no history of fever, headache, and vomiting. There was no history of any weakness of limbs, deviation of mouth or slurring of speech. On examination patient was conscious and oriented with vitals stable. Rest of general examination was normal.

**Figure 1.**
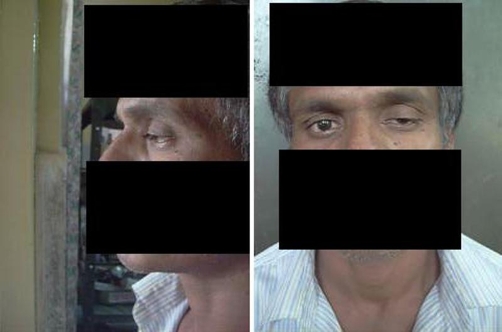
Showing ptosis in left eye in both the views left lateral and front view.

On central nervous system examination higher function were normal. Ptosis and medial rectus weakness of left eye was present (Figure 1), with rest of the movements normal in both eye. There was no proptosis on presentation. Bilateral pupils were normal in size reacting to light. Fundus examination was normal. No other cranial nerves were involved. No neurodeficit was present.

Blood investigations in the form of complete blood count, plasma blood sugar (fasting and postprandial) were normal. X ray chest PA view was within normal limits.

Visual evoked potentials study showed prolonged P100 latency with reduced amplitude of P100 in left eye. Right eye recording was within normal limits.

Ultrasonography (USG) orbit revealed thickened distal part of the left eye medial rectus with increased vascularity with thickened optic nerve with echogenic retro bulbar fat suggestive of inflammation cause. computed tomography (CT) orbit was advised.

**Figure 2.**
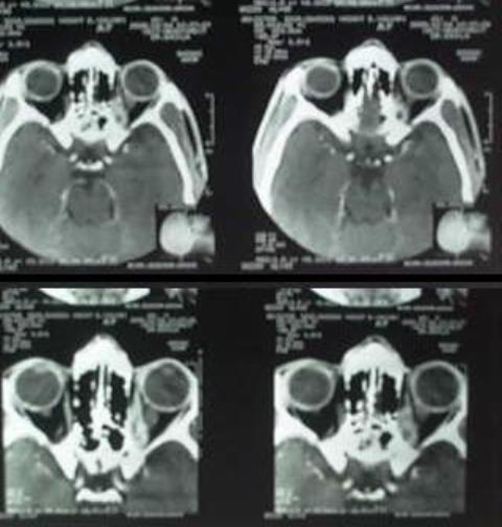
CT orbits showing multiple small non enhancing well defined hypo dense lesion in medial rectus and superior rectus muscles of left orbit.

Computed tomography of brain showed no obvious abnormality. CT orbits revealed multiple small non enhancing well defined hypodense lesion in medial rectus and superior rectus muscles of left orbit posteriorly and the lesion in superior rectus muscle hyperdense dot in central area may likely to cysticercosis causing thickening of the left medial rectus and superior rectus muscles posteriorly comprising optic canal and compressing, thickening of optic nerve (Figure 2).

Accordingly, patient was started on systemic steroids and albendazole in the prescribed doses. On the fourth day of hospital stay patient started responding. Pain subsided, medial rectus palsy and ptosis was partially improved. On 15^th^ day patient turned to the OPD with minimal ptosis and no medial rectus palsy. The movements of both eyeballs were normal in all directions.

## Discussion

Cysticercosis is caused by haematogenous spread and encystment of the larval form of the swine tapeworm *Taenia solium*, in various body tissues. It is the most common parasitic disease of the central nervous system and also affects the eye, skeletal muscle, and subcutaneous tissue.

Soemmering reported the first case of ocular cysticercosis in 18304 [3]. Ocular manifestations may be devastating as the cysticercus enlarges. In the eye cysticerci may be situated intraocular or extra ocular. In India most common site of localization is orbit, whereas posterior segment involvement is more common in western people. Intraocularly, cysticerci occur in vitreous body and sub retinal [4,5] but some may be found in the anterior chamber and subconjunctival [6,7]. The most damaging location is intravitreal and subretinal location which leads to blindness in 3 to 5 years unless the parasite is removed.

It has been pointed out by Malik et al [6] that the left eye is more commonly involved.

Cysticercosis cellulose within the optic nerve is rare. Cysticercosis may cause significant visual loss, especially if the cyst is located in the optic nerve or is compressing the optic nerve. As the condition is often mistaken for optic nerve tumors. On neuroimaging the diagnosis is often delayed or missed. The cyst has a highly reflective pinhead lesion within it representing the scolex, which may be detected by ultrasonography or careful examination of CT or magnetic resonance imaging scans with 1 mm sections.

Intermittent proptosis in our case could be attributed to the inflammation. The predominant symptoms in patients with extra-ocular muscle involvement are proptosis, pain, diplopia, ptosis, and diminution of vision. The most commonest extra ocular muscle to get involved in cysticercosis in medial rectus. Khwaja et al has reported recurrent headache and unilateral ptosis as a manifestation of cysticercosis [8]. Kundra et al has reported a unilateral ptosis due to cysticercosis in a 11 year girl [9]. To our knowledge a manifestation of ptosis, diminution of vision and medial rectus palsy in unilateral eye has been reported for the first time. Therefore, extraocular muscle cysticercosis should be considered in patients who present with restricted ocular motility and inflammatory signs.

The association of brain tissue cysticercosis is very rare with eye cysticercosis [10]. The same is seen in our case. There was no involvement of brain parenchyma in our case.

The treatment of ocular cysticercosis is conflicting. Where the intraocular cyst responds best by surgery, surgical removal of extraocular cysticercosis is fraught with complications. R Sihota et al evaluated the efficacy of oral albendazole in extraocular cysticercosis in the randomized clinical trial and reported a marked clinical respond in the patients [11]. In our case patient was treated with systemic steroids and cysticidal therapy and the response was dramatic.
